# Do Not Fall for This; Diagnostic Challenges in Orbital Floor Fractures With Extraocular Muscle Entrapment

**DOI:** 10.7759/cureus.35268

**Published:** 2023-02-21

**Authors:** Savannah Kumar, Anna Artymowicz, Joseph Muscente, Roman Shinder, David Mostafavi

**Affiliations:** 1 Ophthalmology, New York Medical College, Valhalla, USA; 2 Ophthalmology, State University of New York Downstate Health Sciences University, New York, USA; 3 Ophthalmology, Richmond University Medical Center, Staten Island, USA

**Keywords:** ophthalmology, extraocular muscles, oculocardiac reflex, entrapment, orbital fracture

## Abstract

Extraocular muscles that are entrapped in orbital fracture sites require emergent surgical treatment. Muscle entrapment can present with subtle findings or mimic other conditions, contributing to delays in diagnosis. Here, we present two cases of extraocular muscle entrapment that were not immediately identified. By discussing the diagnostic challenge in these cases, we aim to increase the comfort of all physicians in identifying muscle entrapment in the emergency department.

## Introduction

Orbital fractures are a common injury in patients presenting to emergency departments in the United States, with an estimated 350,379 emergency department visits in the US and a cost burden of more than 2 billion dollars between 2006 and 2017 [1]. When extraocular muscles or associated soft tissue become trapped in an orbital bone fracture, the entrapment can lead to bradycardia, permanent diplopia, or even death. Extraocular muscle entrapment requires urgent surgical correction, making it essential to avoid any delays in diagnosis [2]. As the diagnosis of extraocular muscle entrapment is primarily clinical, a thorough physical examination of the patient is vital to the workup. 

Assessing for extraocular muscle entrapment can fall outside the comfort zone of many physicians involved in emergency care, including ED physicians and radiologists. This article aims to bring awareness to some of the challenges in the diagnosis by sharing the clinical course of two patients in whom the diagnosis of entrapment was initially missed. We aim to increase physician confidence in diagnosing extraocular muscle entrapment in the emergency setting, thus optimizing the quality of care for patients.

## Case presentation

Case 1 

**Figure 1:**
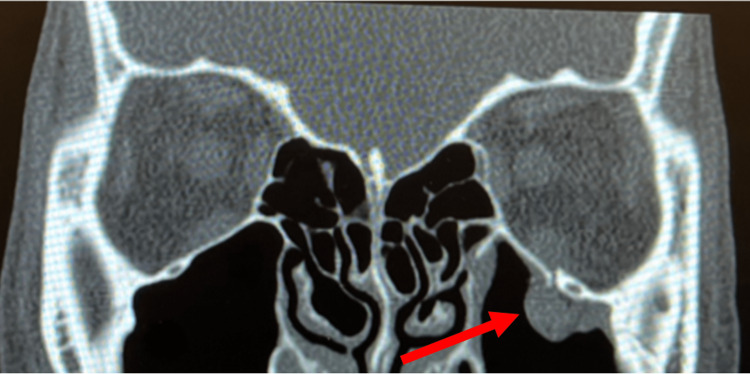
A coronal view of the non-contrast CT (soft tissue window) showing a right-sided orbital floor trapdoor fracture with associated orbital soft tissue entrapment in the fracture (red arrow).

A healthy 12-year-old male with no past ocular history presented to the emergency department after a physical assault in which he suffered blunt trauma to the face. The patient denied vision changes. The physical examination in the emergency department noted edematous left upper and lower eyelids but otherwise normal eye exam with total extraocular motility. The patient's heart rate was found to be 43 beats per minute. A non-contrast computed tomography (CT) scan of the head was performed (Fig 1). The radiology read did not note any facial fractures but a potential mucous retention cyst in the right maxillary sinus. The patient was discharged to home with primary care follow-up. Five days later, the patient reported left eye pain to his pediatrician, who sent him to the emergency department for an urgent ophthalmology consultation.

At that time, an ophthalmic examination of the left eye revealed an inability to supraduct the eye above the horizontal midline, pain on infraduction, and V2 hypoesthesia. The CT scan obtained 5 days prior was reviewed again. The read was revised to suggest possible entrapment of the inferior rectus muscle or associated soft tissue into a fracture at the orbital floor. The patient underwent emergent surgical release of entrapped tissue and repair of the orbital fracture with the placement of an orbital implant on the same day with immediate improvement in supraduction of the left eye. The patient was subsequently discharged home. At the time of discharge, the patient had recovered a full range of extraocular muscle movement in the left eye. 

Case 2

**Figure 2:**
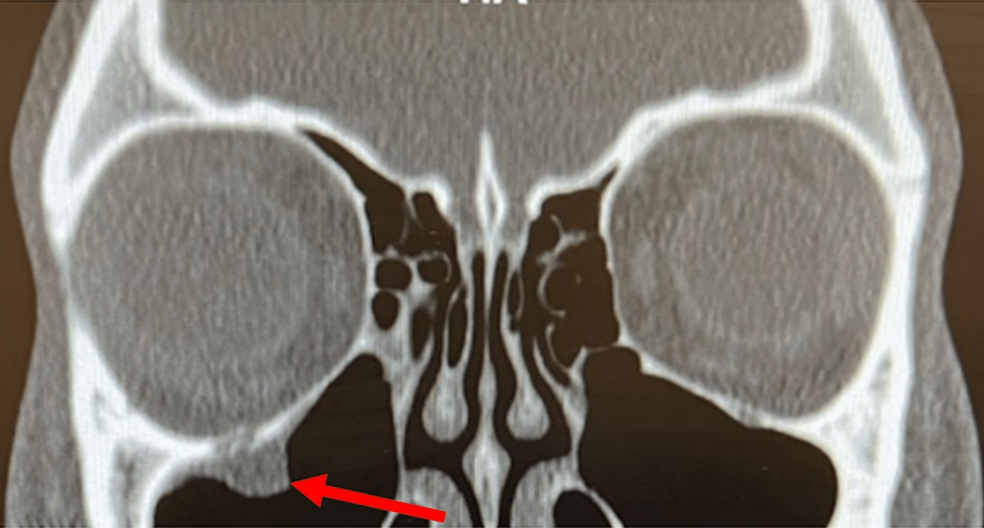
A coronal view of the non-contrast CT (soft tissue window) showing a left-sided orbital floor trapdoor fracture and associated orbital soft tissue entrapment in the fracture (red arrow).

A healthy 20-year-old female with no past ocular history presented to the emergency department four days after an assault during which she sustained high-energy blunt trauma to her face. The patient reported right eye pain, double vision, blurry vision, and headache. Vital signs were within normal limits, and the external exam was unremarkable, without edema or ecchymosis of the face. All extraocular movements were noted as intact and painless. A maxillofacial CT scan was obtained and interpreted by radiology as a "fracture of the right orbital floor with herniation of fat into the right maxillary sinus" (Fig 2). The patient was discharged to home with outpatient follow-up with ophthalmology. On evaluation by ophthalmology the following day, the patient was found to have limited supraduction of the right eye associated with pain, consistent with entrapment. The rest of the examination was within normal limits. The patient underwent surgical release of entrapped tissue and repair of the orbital fracture with the placement of an orbital implant the next day. Two months after the surgery, the patient recovered full extraocular motility and reported complete resolution of her diplopia. 

The two cases above illustrate common challenges in diagnosing orbital fractures with entrapment in the emergency setting. In the first case (case #1), the trapdoor fracture with entrapment was misinterpreted as a mucous retention cyst, bradycardia was not identified as a potential sign of entrapment, and the clinically limited eye movements were not noted until outpatient follow-up. In the second case (case #2), the CT scan was interpreted as an orbital floor fracture with tissue herniation. However, the external exam did not show significant edema or ecchymosis, which led to an inappropriately low clinical suspicion of entrapment. In this case, the patient's eye movements were erroneously judged as complete until outpatient follow-up. 

Both cases highlight the importance of careful eye examinations in patients with orbital fractures and the necessity for clinical correlation with radiographic imaging. Thankfully, both patients underwent prompt treatment after entrapment diagnosis and fully recovered extraocular muscle function. However, not all patients have a prompt or reliable follow-up with specialists after their initial care. Missing the diagnosis of entrapment in the emergency room could expose the patient to undue risk of permanent disability or even death.

## Discussion

Orbital fractures - Etiology 

Bones of the orbit are vulnerable to fracturing on experiencing significant direct force. Orbital fractures are commonly associated with assault, falls, traffic accidents, or sports injuries and are more common in younger and male patients [3,4]. The orbit is conceptually divided into four walls- the roof, floor, lateral, and medial walls. The most common orbital wall to fracture is the orbital floor, followed by the medial wall [3,5]. These are particularly vulnerable locations as the orbital floor is relatively unsupported by the maxillary sinus, while the medial orbital wall contains the thinnest bones of the orbit [6]. 

Muscle and soft tissue entrapment happen when a fractured bone displaced by the fracturing force moves back towards its normal non-displaced position, trapping tissue into the fracture site. This so-called trapdoor style fracture is more common in pediatric patients due to the more malleable nature of younger bone [7-9], but it has also been reported in adults [10,11], including our patient in case #2. The symptoms most commonly reported by patients with extraocular muscle entrapment are diplopia and pain with eye movements (87.5%), nausea or vomiting (37.5%), and a heart rate of 60 bpm or less (50%) [12]. 

Ophthalmic examination of patients with an orbital fracture is often significant for periorbital edema and ecchymosis. However, it is important to recognize a phenomenon known as white eye blowout fractures, as illustrated in case #2. These fractures are characterized by a benign external appearance with underlying entrapped tissues [2] and are more common in pediatric patients. The "white" appearance of the eye is attributed to the minimal soft tissue trauma in these injuries and, thus, the minimal ecchymosis and swelling observed in these injuries. The benign appearance of the eye can lead to a false sense of security, potentially causing a delay in the diagnosis of entrapment which can lead to significant consequences.

Sequelae of extraocular muscle entrapment - Why do we care?

Entrapped soft tissue not surgically released promptly can become ischemic, leading to contracture and compromised muscle function even after surgical release. The resultant asymmetry in extraocular muscle function can lead to persistent diplopia, which profoundly affects the quality of life [13] and may necessitate subsequent corrective surgery of extraocular muscles.

As the early intervention is associated with better postoperative function, it is generally recommended that entrapped fractures be repaired within 48 hrs of the insult [14,15]. If there is an associated arrhythmia resulting from the oculocardiac reflex, intervention should be as soon as possible (within hours).

Even before ischemic damage occurs, a potentially life-threatening consequence of extraocular muscle entrapment is bradycardia or asystole caused by the oculocardiac reflex, also known as the Dagnini-Aschner phenomenon [16]. A triad of bradycardia, syncope, and nausea characterizes the oculocardiac reflex. The connection mediates it between the ophthalmic branch of the trigeminal nerve, and the vagus nerve is stimulated by the traction of extraocular muscles [9,17]. In the setting of entrapment, this can be triggered when a patient attempts to look away from the entrapment site. Though the reflex often results in inconsequential sinus bradycardia, it has been reported as the cause of cardiac arrest, arrhythmia, and asystole [7,18].

Extraocular muscle assessments - How to assess?

Extraocular movements (EOM) may be hard to assess in children, in uncooperative patients, in those with significant facial soft tissue edema or lacerations, or in those with significant chemosis [6,8]. Utilizing the help of an assistant to open the eyelids during the extraocular movement exam can be helpful. The patient should be asked to look in all four cardinal directions (up, down, left, right) to the maximum degree possible. Scleral show and reliance on the gestalt appearance of the eye are unreliable in assessing eye movements. A reliable way of assessing vertical limitations in eye movements is to draw an imaginary line across the most inferior portion of the limbus of one eye and compare the two eyes. A reliable way to assess horizontal limitations in eye movements is by observing the corneal reflex from a light source centered on the face at least one arm's length away. Eyes that are aligned will reveal light reflexes in the corresponding part of the cornea. 

Interpreting the EOM exam can be difficult. Though new ocular misalignment after trauma (noted in any direction of gaze) should raise suspicion for muscle entrapment, preexisting comorbidities, and periocular edema can muddle the clinical picture. It is, therefore, valid for the clinician to remember specific signs that further raise suspicion for entrapment. For example, diplopia on gaze directed away from an orbital fracture site or diplopia both towards and away from the fracture site, dubbed "double diplopia," are both commonly associated with entrapment. Pain or an increase in the intraocular pressure of greater than 4 mm when attempting to look in the diplopia field are other phenomena that raise suspicion for entrapment over other potential causes of EOM restriction [13].

To assess for the oculocardiac reflex, the patient should be connected to a heart monitor. The baseline heart rate should be noted on the primary gaze/ with eyes at rest. The reflex is considered positive if the heart rate decreases by at least 20% within the first 5 seconds of attempting an extreme gaze [17]. The reflex will usually be positive when looking in the direction away from the entrapment. We suggest asking the patient to maintain an extreme gaze in each of the four cardinal directions for 10 seconds each to allow sufficient time for the heart monitors to pick up any change in heart rate.

Imaging - Potential challenges

Suboptimal imaging can make the identification of orbital fractures challenging. A computerized tomography head scan, typically ordered for trauma cases involving the head, may not encompass the orbits and is taken in slices too thick to identify small orbital fractures [2]. Orbital or maxillofacial scans (which will obtain thin slices through the orbits) are recommended when there is suspicion of facial or orbital fractures.

Trapdoor fractures (where the fractured bone moves back into its non-displaced position, trapping tissue at the fracture site) are especially challenging to identify as radiographic evidence of a closed fracture may be minimal, even with optimal imaging protocols [2]. This leads to an underestimation of soft tissue entrapment by a radiographic read of a CT scan alone. This discrepancy is most noticeable in the pediatric population, where the radiologist estimated that 50% of entrapped fractures could go unappreciated [19]. It is, therefore, paramount to remember that the diagnosis of entrapment is clinical and be wary of normal CT reads or alternative explanations for abnormal CT periorbital findings in the setting of clinical suspicion for entrapment.

## Conclusions

Our cases illustrate typical challenges in diagnosing muscle entrapment in orbital fractures. Effectively identifying restrictions in eye movements, clinically correlating radiographic findings, and assessing for an oculocardiac reflex can help prevent delays in diagnosis. Delays in treatment can lead to lifelong consequences. Thankfully, both patients described in this paper were diagnosed on follow-up and successfully treated. 
